# Addressing Health Inequalities in the Delivery of the Human Papillomavirus Vaccination Programme: Examining the Role of the School Nurse

**DOI:** 10.1371/journal.pone.0043416

**Published:** 2012-09-13

**Authors:** Tammy Boyce, Alison Holmes

**Affiliations:** National Centre for Infection Prevention and Management, Department of Medicine, Imperial College London, Hammersmith Hospital, London, United Kingdom; The University of Hong Kong, Hong Kong

## Abstract

**Background:**

HPV immunisation of adolescent girls is expected to have a significant impact in the reduction of cervical cancer. UK The HPV immunisation programme is primarily delivered by school nurses. We examine the role of school nurses in delivering the HPV immunisation programme and their impact on minimising health inequalities in vaccine uptake.

**Methods and Findings:**

A rapid evidence assessment (REA) and semi-structured interviews with health professionals were conducted and analysed using thematic analysis. 80 health professionals from across the UK are interviewed, primarily school nurses and HPV immunisation programme coordinators. The REA identified 2,795 articles and after analysis and hand searches, 34 relevant articles were identified and analysed. Interviews revealed that health inequalities in HPV vaccination uptake were mainly related to income and other social factors in contrast to published research which emphasises potential inequalities related to ethnicity and/or religion. Most school nurses interviewed understood local health inequalities and made particular efforts to target girls who did not attend or missed doses. Interviews also revealed maintaining accurate and consistent records influenced both school nurses' understanding and efforts to target inequalities in HPV vaccination uptake.

**Conclusions:**

Despite high uptake in the UK, some girls remain at risk of not being vaccinated with all three doses. School nurses played a key role in reducing health inequalities in the delivery of the HPV programme. Other studies identified religious beliefs and ethnicity as potentially influencing HPV vaccination uptake but interviews for this research found this appeared not to have occurred. Instead school nurses stated girls who were more likely to be missed were those not in education. Improving understanding of the delivery processes of immunisation programmes and this impact on health inequalities can help to inform solutions to increase uptake and address health inequalities in childhood and adolescent vaccination programmes.

## Introduction

Cervical cancer, like other cancers, disproportionately affects poor women. A meta-analysis of global trends found an estimated 100% increased risk of invasive cervical cancer for women in low social class categories compared to those in higher social class categories [1]. In the UK incidence and mortality from cervical cancer is strongly related to deprivation [2], [3], [4], [5]. The cytological screening programme has reduced the number of women who die from Human Papillomaviruses (HPV), the cause of cervical cancer, however there are persistent health inequalities in this screening programme; poor women have lower attendance rates than wealthier women [6], [7]. The HPV vaccine was introduced as a primary prevention strategy to reduce the incidence of cervical cancer. Many countries offer the HPV vaccine as part of their national immunisation programme including; the UK, Australia, Canada, France, Greece, New Zealand, Norway and Sweden. In many of these countries the HPV vaccination programme is delivered by school nurses (for example; in the UK, Australia, Canada, Norway, Sweden). This research examines the role of the school nurse in the delivery of this routine adolescent vaccination in the UK and explores how their efforts to deliver the HPV vaccine affects health inequalities.

**Figure 1:**
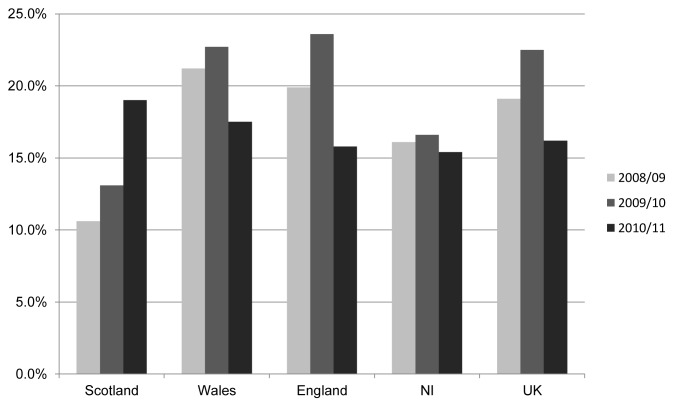
Girls not fully vaccinated with 3 doses of HPV vaccine in UK 2008–11.

The HPV immunisation programme is expected to have a significant impact on public health. It is estimated that the programme will reduce deaths from cervical cancer by two-thirds if uptake reaches 80% [8]. Since it was introduced in September 2008, uptake of the HPV vaccine in the UK has been high compared to other European countries [9], [10]; leading the Department of Health in England to describe the first two years of the HPV programme as a [success] [8], [11]. The HPV vaccination programme has achieved high uptake in some areas in the UK, however a significant number are not vaccinated or do not complete the full vaccination schedule (See Figure 1 [11], [12]).

Figure 1 shows the many missed individual doses but HPV immunisation uptake also varies by locality, suggesting there are health inequalities in uptake. Within the city of Birmingham uptake of the HPV vaccination reflects the burden of deprivation. Uptake is lower in the city centre where deprivation is concentrated, rises slightly in the East and North where there is slightly less deprivation and is highest in the South, where there are lower levels of deprivation [13] (See Table 1 [11]). Low uptake of childhood and adolescent vaccines has been linked to deprivation, ethnicity, single parenthood and residence in areas with high levels of diverse ethnicity or low income [14], [15], [16], [17], [18], [19], [20], [21].

**Table 1:** HPV vaccination coverage in Birmingham.

2010–2011 HPV vaccine coverage in Birmingham, England (%)			
Strategic Health Authority range	Dose 1	Doses 1&2	All 3 doses
South Birmingham PCT	92.8	92.1	88.9
Birmingham East and North PCT	80.8	80.1	77.9
Heart of Birmingham PCT	75.1	74.2	71.3

In England only one of the ten local authorities with the highest estimates of child poverty [22] achieves over 90% HPV vaccination uptake (Blackpool. Of these ten local authorities, representatives from seven areas were interviewed for this research). Five of the areas with the highest rates of child poverty have a mean HPV vaccination uptake below 70 per cent (Hackney, Westminster, Camden, Haringey, Barking and Dagenham). Little research has examined inequalities in HPV uptake. One piece of research concludes that where the HPV vaccine is delivered is the [root cause of disparity] as school-based programmes consistently achieved higher uptake than the catch-up cohort, (which was mainly delivered in General Practices) [23].

The HPV vaccine was introduced during an era of increasing attention to health inequalities in all four UK home nations [24]. In each nation policies emphasise the importance of addressing the social determinants of health to reduce inequalities however, in the quest to address these social determinants, public health interventions (such as vaccination programmes) should not be forgotten as effective methods to reduce health inequalities: if delivered in an equitable way. Identifying specific interventions to reduce inequalities is often difficult [25], [26]. Compared to other interventions, vaccination programmes are easier to monitor for inequalities and have the potential to be a valuable part of a public health programme to reduce inequalities. This research provides evidence of the capacity and ability of school nurses to address inequalities in the delivery of the HPV vaccination programme, whose role in addressing inequalities is poorly understood [27].

## Methods

This research is based on a rapid evidence assessment (REA) followed by interviews with health care professionals. Mixed methods provide rigorous and methodologically sound research [25], [28], [29], [30], [31]. The REA sought to identify key themes to be discussed in the interviews. The interviews aimed to confirm or challenge existing findings and identify additional and as yet unidentified issues related to the delivery of the HPV vaccine programme and health inequalities.

### School Nurses in the UK

As health is a devolved responsibility in the UK, each of the four home nations has a different school nursing policy. The majority of school nurses are employed by Primary Care Trusts (in England) or Local Health Boards (in Scotland, Wales, Northern Ireland). In Wales, since 2011, each comprehensive school has its own school nurse employed for 52 weeks of the year. Outside Wales, many school nurses cover more than one comprehensive school and are employed only during term time [32]. Northern Ireland policy recommends all primary and secondary schools have a dedicated school nurse but it is unclear how many schools each school nurse covers. Similarly, the 2003 school nursing framework in Scotland recommended one school nurse to cover a cluster of schools, but due to lack of evidence it is unclear how many schools school nurses cover. In England the Coalition Government declared their support for school nursing in March 2012 [33]. This report stated school nurses have an important role in providing additional services to vulnerable children and their families. The number of school nurses in England increased between 2004 and 2008 however between September 2009 and April 2010 the number of full-time equivalent school nurses fell by 9.1 per cent [34]. The Royal College of Nursing argues [minimum] numbers of school nurses may not meet the need in many areas and that [(t)he Government should ensure that money is available to fund additional posts if an effective service is to be provided] [35].

### The REA

A REA uses methods from a comprehensive systematic review but the timescale is shortened by focussing the research question, using broad search strategies and restricting the amount of grey literature analysed [36]. This REA includes a sample of grey literature as it involved searching OpenSigl, a portal for grey literature. It also includes documents from interviewees. Several researchers argue that extensive literature reviews introduce bias [as trials that are harder to locate can be of lower quality] [37]. REAs analyse higher quality evidence in peer-reviewed texts rather than spending additional time searching for more obscure references [38].

Nine electronic databases were searched: PubMed, Science Direct, Social Science Research Network, Web of Knowledge/Science, Embase, National Library for Public Health, NHS Evidence, OpenSigl and the York Centre for Reviews and Dissemination. Advice was taken from specialist librarians to define research terms in different databases. Terms were amended for each database. A ‘*’ is used when searching databases to include terms with different suffixes, so ‘vaccin*’ includes ‘vaccine/s’ and ‘vaccination’. The search terms used were: ‘HPV vaccin*’, ‘inequal*’ and ‘socioeconom*’. To widen the possibility of findings articles related to health inequalities, the search also included the following: ‘ethni*’, ‘lesbia*’, ‘homosexua*’, ‘learning dif*’, ‘mental di*’, ‘prostit*’, ‘refugee’, ‘asylum seeker’, ‘homeless’, ‘prison*’, ‘offend*’ and ‘gypsy’. These terms were based on the groups included in the Cabinet Office report ‘Inclusion health: Improving the way we meet the primary health care needs of the socially excluded’[39].

Each abstract was read to analyse if it met the inclusion criteria. The REA inclusion criterion was broad, all articles were included (e.g. editorials, letters) and there was not a date restriction. The final search was conducted in September 2011. Articles were included if they related to the UK and analysed the HPV vaccine in relation to health inequalities. Studies and articles were excluded if they were not UK based, were not published in English or were a clinical trial with an HPV vaccine. If the abstract lacked information to assess its inclusion, the full paper was read. A total of 2,795 articles were identified and relevant articles entered into a database.

### The Interviews

Health professionals who deliver the HPV immunisation programme across the UK were interviewed between June and August 2011. The interviews focused on their role in the HPV programme and their efforts to address health inequalities (see Box S1). Two methods of sampling were used; convenience sampling and snowballing. The Royal College of Nurses and the School and Public Health Nurses Association were contacted and agreed to send an email to school and practice nurses outlining the research and a request to be interviewed. The aim of the convenience sample was to interview school nurses from a range of areas across the UK, including areas of high deprivation. Sampling did not seek to be representative but to reflect diversity within the group [40].

Snowballing techniques were then applied; interviewees were asked to suggest others who might be willing to be interviewed or provide alternate or innovative examples of addressing health inequalities. This purposive sampling technique sought to achieve wider representation and to include special or unique cases [41].

80 health professionals were interviewed. Extensive efforts were made to interview health professionals from each of the four home nations, rural and urban areas and areas of deprivation. The decision to stop interviews was made when thematic saturation was reached (when new themes did not arise) and when an appropriate range and geographical representation of health professionals from across the UK were interviewed [42].

71 interviews were held over the telephone and notes recorded. The use of note-taking (instead of recording) may introduce a risk of bias but it was a deliberate decision to take notes as this forces the researcher to concentrate more closely [43]. In addition, recording interviews can [alter] conversations and create [particular contexts for what is said] [44]. Often the default is to record qualitative interviews, however there was concern that as the interviews aimed to be short, there would not be time to build up trust between the interviewer and interviewee or time to discuss permission to record the conversation. The interviews were semi-structured, based on open-ended questions and typically lasted 15–20 minutes. Nine interviews took place over email. These email interviews included detailed descriptions of their services and an exchange between the author and interviewee covering questions in the topic guide. All interview participants were informed of the purpose of the research and that notes were being recorded and assured their comments would be anonymised.

Interviews were analysed using a two-level systematic thematic analysis [45], [46]. A list of deductive codes was initially created. Inductive codes emerged during the second level of the thematic analysis and findings from the REA also helped to create these codes [47].

Direct quotations have been edited to amend minor grammatical errors.

Interviewees are described using the 2010/2011 HPV vaccine uptake.

## Results

### Rapid Evidence Assessment

A total of 27 articles were initially identified using the inclusion criteria. All of these articles were entered into a database. Snowballing techniques (analysing references in the bibliographies and further hand searches) were then completed to identify other high quality articles [48], leading to seven more articles. 34 articles were included in the REA. Of the 34 articles there were: 23 original research articles, five reviews, three editorials/analysis and three letters (Two letters to the editor involved original research but remain labelled as ‘letters’ as they would not have been put through a peer review process similar to an original research article). The majority of the original research, 68%, is quantitative analysis (primarily large scale surveys). The surveys included 16,744 participants and 1327 schools.

### Interviews

62 school nurses were interviewed between June–August 2011, the remaining 18 health professionals included practices nurses, administrators, civil servants, a health visitor and a pharmacist. 55 interviewees were from England, 15 from Scotland, 9 from Wales and 1 from Northern Ireland (See Table 2).

**Table 2:** Health professionals interviewed.

	Total	England	Scotland	Wales	N. Ireland
School nurse	36	22	9	5	
Coordinator HPV immunisation programme (most school nursing background)	26	23		2	1
Practice nurse	7	6	1		
Administrator	5	2	1	2	
Civil Servant	2		2		
Health visitor	1	1			
Lead public health nurse	1	1			
Pharmacist	1		1		
Public health doctor	1		1		

### Thematic Analysis

The thematic analysis, based on findings from the REA and the interviews, identified three key themes concerning health inequalities and the HPV vaccination programme; (i) variations in delivery (ii) actual inequalities in the HPV vaccination programme and (iii) accuracy in record-keeping.

### (i) Delivering the HPV vaccination programme

Little research examines the structure or delivery of the HPV immunisation programme in the UK. Since the REA was completed, one article has examined school nurses' experiences of delivering the HPV vaccination [49]. The article found the HPV vaccination programme increased school nurses workload and that school nurses were concerned that they had less time to support vulnerable students. It is unclear from this article if ‘support’ is in relation to the HPV programme or in associated with wider health concerns. This research only interviewed 30 school nurses during the first year of the HPV immunisation programme whereas our research interviews 80 health professionals, including 36 school nurses and 26 co-ordinators (most of whom were school nurses) and interviewed them during the third year of the programme.

Each area in the UK was responsible for designing and delivering their HPV immunisation programme. The majority chose to follow the recommendation from the Joint Committee on Vaccination and Immunisation to immunise the routine cohort in schools with school nurses. (In the first three years of the HPV vaccination programme an additional ‘catch-up’ campaign targeted girls aged from 14 to 18 years. Each area also decided how to vaccinate the ‘catch-up’ cohort and to make arrangements for those who missed a scheduled immunisation. The ‘routine’ cohort and ‘catch-up’ cohort were vaccinated in schools and there were also efforts to vaccinate the ‘catch-up’ cohort in general practice.) School nurses described that a typical school-based HPV vaccination of the routine cohort involved a number of opportunities for girls to be vaccinated;

Typical delivery of HPV vaccine schools-based programme

1. School nurses work with school to arrange three dates for vaccination sessions.
2. School nurses deliver health promotion session and distribute consent forms. Most sessions are done in assemblies and include boys and are held in the autumn. Some are held in summer term.
3. Dose 1 (September/October).
4. Dose 2, Dose 1 Mop-up clinic (before Christmas). (Mop-up clinics were held to vaccinate girls who missed a dose. Catch-up clinics were offered during the first three years of the HPV programme to target older girls.)
5. Extra Mop-up clinic (before Christmas).
6. Dose 3, Dose 2 Mop-up clinic (before Christmas).
7. Mop-up clinic (After Christmas).
8. Additional/Weekly mop-ups clinics/home visits (After Christmas).

In some areas, simply employing a school nurse increased uptake; ‘Now that we have a school nurse, we are going flat out to get each girl whereas before just did those with consent forms’ (South West Scotland, achieving over 90 per cent uptake all three doses [55]).

The frequency of mop-up clinics and where they were held reflects the efforts of school nurses to address health inequalities. School nurses who organised mop-up clinics that suited the girls rather than themselves appeared to have more success. Most mop-up clinics for the routine cohort were held in the local school or Health Centre but for girls not in school or poor attenders, mop-up clinics that were held off school premises or outside school hours appeared to improve attendance. One area covering a large rural area offered mop-up clinics in the main city's concert hall on a Saturday afternoon as they believed it would ‘accommodate more girls, taking into consideration the time they might be up and about, the attraction of shopping and the access for young women who may have had Saturday work in the city’ (Central Scotland, achieving over 90 per cent uptake all three doses). Another area immunised in a local sports centre where social workers brought girls who had not been immunised (South West Scotland, achieving over 90 per cent uptake all three doses). One school nurse acknowledged where mop-up clinics were organised did affect uptake and as such, they were moving their mop-up clinics from a children's centre as ‘many older girls do not like to attend the clinic there’ (North West England achieving just over 80% first two doses). Considering the needs of young girls demonstrates the flexibility and sensitivity of some school nurses who used their knowledge and experience to initiate innovative solutions to improve uptake and minimise inequalities.

### (ii) Expected versus ‘actual’ inequalities

#### Religion and ethnicity

The UK HPV immunisation programme has been in place for over three years yet research is dominated by ‘anticipated’ inequalities. Research carried out before the launch of the HPV immunisation programme hypothesised that those with significant religious beliefs would be less likely to accept the vaccine [50], [51], [52]. 54% of the articles in the REA analysed potential inequalities related to ethnicity and/or religion [15], [50], [51], [52], [53], [54], [55], [56]. In many of these articles the authors admit the effect sizes of ethnicity are too small or there are lower degrees of confidence in statistical findings due to the small numbers studied [15], [50], [54], [55], [57], [58]. Regression analysis of HPV vaccination uptake found ethnic composition dominated the explanation of uptake only in the routine cohort [23].

In contrast to the published research, interviews with school nurses stated in their experiences religion and ethnicity had little effect on HPV vaccination uptake. School nurses stated uptake was sometimes lower in schools with a large religious population and that uptake in some Muslim and Catholic schools was low. In a very small number of these schools school nurses struggled as head teachers in religious schools decided not to offer the HPV vaccine (East of England; South Wales). However in many other areas school nurses stated they had good uptake in schools with high percentages of Muslim or Catholic students or religious schools (North London, Central London).

In many areas school nurses reported religious leaders had a significant impact on the uptake of the HPV immunisation programme, either in encouraging or rejecting the vaccine. In one area where uptake was low in a school with a large Muslim population, school nurses made links with the local Imam and Muslim leaders. In a few areas head teachers of Catholic schools told parents and girls that the Pope supported the HPV vaccine and school nurses stated this increased uptake (Central England; North East England, both achieving over 88% uptake first two doses). Support from religious leaders was not consistent, even within the same religion. In another area uptake in the Catholic school was low and school nurses said the local priest and head teacher had rejected the vaccine (North West England, achieving just over 80% first two doses).

Previous research concentrated on potential problems resulting from Muslim or Catholic religions however school nurses reported that smaller religious groups rejected the HPV vaccine. School nurses stated smaller religious schools, such as Christian schools, a Church of Wales school and ultra-Orthodox Jewish schools largely or entirely rejected the HPV immunisation programme. Areas with substantial ultra-Orthodox Jewish populations found almost all of these girls rejected the HPV vaccine (North London, North West England), reflecting research that finds low uptake of other childhood vaccinations in this community [59]. School nurses hesitated in promoting the HPV vaccine in these schools as the ‘difficulty is if you push they will put walls up and won't accept other vaccines’ (North West England achieving just over 80% first two doses).

#### The actual inequalities

When asked who was likely to miss the HPV vaccine, many school nurses quickly stated they knew who would be difficult to vaccinate - vulnerable girls; ‘you know the ones that don't attend, we send 5 or 6 letters…For those that did not attend, we keep giving them chances’ (South Wales, achieving over 95% uptake all three doses). Other school nurses had similar levels of awareness of those who were regarded as ‘hard to reach’; ‘nurses know the names’ (South West Scotland, achieving over 90 per cent uptake all three doses). Another school nurse agreed that the ability to identify the vulnerable girls was important; ‘it's about knowing case load…we target vulnerable kids, it's difficult, you need to be prepared that you'll be challenged but it is more enjoyable and valuable because their health needs are grim’ (North Wales, achieving just over 89% first two doses).

#### Girls with learning difficulties, travellers and ‘Looked After Children’

School nurses described how they identified and targeted a number of vulnerable groups. For example, most school nurses made efforts to vaccinate girls with learning difficulties. Girls with special needs are almost entirely absent from previous research into the HPV vaccine, in one article a school nurse states she found it difficult to vaccinate this group as [they get very agitated and keep walking off] [60] but this issue is not further explored. As these girls have complex medical needs, school nurses spent more time convincing parents the vaccine was needed. They did this by creating a trusting relationship with both girls and their parents; ‘It also takes longer to get trust and convince girls it is ok’ (Central England, achieving over 78% first two doses), ‘parents think the HPV vaccine is unnecessary as they will not be sexually active’ (North East England, achieving over 88% first two doses). These girls were often vaccinated in special needs schools or at home.

Establishing trust and having a flexible attitude was also important when vaccinating travellers and gypsies, a group with poor health and low uptake of childhood vaccines [61], [62], [63], [64]. Many areas used the same health care worker (either a school nurse, nurse or health visitor) who had an established relationship; ‘Word of mouth worked in my favour’ (school nurse who vaccinated 16 travellers in 2009/10). Another nurse worked with the local school to identify girls from the travelling community and immunised on site (North Scotland, achieving over 91% first two doses). One nurse said she delivered the HPV vaccine ad hoc to girls in the traveling community and vaccinated; ‘when she can, not according to the programme. It's hit or miss’ (North Wales, achieving over 89% first two doses). When one area offered the HPV vaccine off-site after giving it at home, they had a zero uptake (South Wales, achieving over 82% first two doses).

Another vulnerable group school nurses made efforts to vaccinate were girls held in custody or in the care of social services. Many school nurses made additional efforts to vaccinate these girls, describing them as ‘the most vulnerable girls and (I want to) ensure they get them’ (South West Scotland, achieving over 90 per cent uptake all three doses). To vaccinate these girls, school nurses typically worked in partnership with youth offending team nurses or secure units and local ‘Looked After Children’ nurses to vaccinate girls in the care of social services.

#### Girls not in school

Instead of religion or ethnicity affecting HPV vaccination uptake, school nurses stated girls who were likely to miss doses of the HPV vaccine were those not in school. Only one article in the REA briefly considered how health care professionals target girls not in school or poor attenders, stating that [students with poor attendance may be underrepresented] [65]. There was a mixed response and understanding of how to target girls not in school. Typically, areas with high HPV vaccine uptake regarded girls not in school as part of their schools-based programme. For example, in an area that reaches over 90% for the first two doses, the immunisation team worked with social workers to target homeless teenagers. Another school nurse ‘liaised with homeless nurses and did drop-in sessions for those educated out of school’ (South West Scotland, achieving over 90 per cent uptake all three doses).

Not all school nurses were knowledgeable about potential inequalities or made efforts to vaccinate girls not in school. One school nurse's knowledge of her local travelling community explains how some vulnerable girls are missed, stating she had not ‘gone out to target travellers specifically. We could do, but if they're in school they are offered it.’ (South London, achieving under 80% all three doses). Other school nurses did not consider how to vaccinate those not in school; ‘We do not currently have a programme for pupils not in school as we are a school based service’ (Central England, achieving under 80% all three doses). This group of school nurses described girls not in school as ‘hard to reach…difficult to find’ (North London achieving under 63% all three doses), ‘unless we see them in school it's very difficult’ (South London, achieving under 80% all three doses).

### (iii) Accurate and persistent records

The number of girls to vaccinate in each school is based on lists provided by the local education authority (LEA) (or its equivalent). Many school nurses from across all four nations stated the information LEAs provided was frequently wrong or not up to date. One school nurse was frustrated with the lists she received from the LEA, describing them as ‘three months out of date’ (North West England, achieving under 85% all three doses). Nurses from different areas said data from the LEA was often slow to be delivered, ‘We want Year 7 in July but sometimes don't get until girls are already in Year 8’ (North West England, achieving under 85% all three doses). A common frustration was that atabases were not updated. One nurse said the LEA supplied them with out of date data and that, ‘incredibly…(they) had the exact same number of girls over three years’ (South West England, achieving under 85% all three doses). In addition, the type of information LEAs offered was inconsistent across the UK. For example, in some areas the LEA provided school nurses with addresses of those not in school but in other areas they would not provide these addresses and instead sent invitation to vaccinate letters on behalf of nurses; leaving school nurses unaware if and/or when letters were sent.

Administrative staff were frequently identified as valuable members of the immunisation team that helped school nurses maintain accurate records and as a result, minimise inequalities. Administrators also spent time persistently chasing girls to complete all three HPV vaccine doses. Without administrative support many school nurses, particularly in areas where there are many girls to chase (i.e. in areas of high deprivation or with large numbers of girls from vulnerable groups), school nurses stated they were likely to struggle to achieve higher uptake rates or eradicate health inequalities. One school nurse described the reason for their high uptake; ‘School nurses couldn't meet need alone. Teams go into schools and blitz each school. The school nurse and health care assistant help along with clerical assistance’ (South West Scotland, achieving over 90 per cent uptake all three doses). One of the consistent themes that surfaced in the interviews was the repeated number of times girls needed to be contacted and that vulnerable girls needed to be contacted more often. Where health professionals were persistent and offered numerous opportunities to be vaccinated, uptake was higher.

## Conclusion and Discussion

As the HPV immunisation programme becomes embedded in the adolescent vaccination schedule it is essential that health professionals and local management bodies of the vaccination programme monitor HPV vaccination uptake to understand and eradicate inequalities. This study is the first to assess inequalities in the delivery of the HPV immunisation programme across the UK. Too often research into health inequalities of vaccination programmes only includes epidemiological analysis of uptake: research should also examine the practices and solutions that have worked to maximise uptake and address health inequalities. Previous research on the HPV vaccine programme concentrates on understanding individual behaviours, primarily analysing the attitudes of parents or girls. The existing research base on specific vulnerable groups provides little information on how to improve uptake of adolescent vaccinations in vulnerable groups.

Our research found delivery of the HPV immunisation programme varied but was fairly similar across the UK with many school nurses being flexible and providing girls with many opportunites to be vaccinated. Many school nurses made substantial efforts to address potential inequalities in the delivery of the HPV programme. They stated girls who were more likely to miss all three vaccines or not complete the three doses were the ‘usual suspects’; coming from areas and communities of high deprivation and from particular vulnerable groups such as travellers. The relationship between income and vaccination decisions is not straightforward as some studies show having *more* education and higher income is associated with decreased likelihood of having the HPV vaccine [66]. In the case of the HPV vaccine, our findings support research that observes those with lower education levels and/or from less wealthy families are less well vaccinated [67], [68].

To minimise inequalities in the HPV vaccination programme, school nurses stated that they often needed additional time to vaccinate vulnerable girls. This additional time was needed to give girls as many opportunities as possible and to develop trust between the girl/her family and the school nurse. School nurses used their own knowledge and experience to identify interventions to vaccinate vulnerable girls as they were offered little guidance from the Department of Health. They actively pursued girls and many stated they were personally motivated to vaccinate as many girls as possible. This finding contrasts with previous research that indicates school nurses were frustrated with large scale immunisation programmes [69], [70] or that the HPV vaccination programme may reduce the time spent with vulnerable pupils to address general health [49]. Instead, this research confirms findings from Sweden [71], where school nurses had positive attitudes towards the HPV programme and believed it could reduce health inequalities. Our findings show that many school nurses regarded the HPV programme as an opportunity to address health inequalities and actively sought to minimise inequalities.

This research also highlights the importance of maintaining accurate vaccination registers. Inaccurate lists affect both uptake statistics and staff morale. Local education authorities need to work with schools and their nurses to ensure lists are accurate. Similarly, if school and practice nurses could better share vaccination history, such as through shared computer systems, this would also ensure more accurate lists.

Policy-makers should be optimistic that reducing inequalities in school based vaccination programmes is possible. School nurses are an untapped resource to address health inequalities: many made substantial efforts and were able to identify and vaccinate difficult to reach girls within existing resources.

### Strengths and weaknesses of the study, future areas of research

Some of these findings may be affected by the fact interviewees were self-selected and may have particular views on vaccination. The aim of this research was to provide an understanding of how school nurses addressed health inequalities in the HPV immunisation programme, it was not to provide a comprehensive four nation analysis of the delivery of the HPV immunisation programme. Another limitation was the lack of publically available data about the HPV programme and uptake in Scotland, Wales and Northern Ireland. In addition, the majority of published research about the HPV programme in the UK analyses the English context therefore reviews and research such as this will inevitably emphasise findings from England. This is unfortunate as HPV vaccination uptake is high in many areas of high deprivation Wales and Scotland, suggesting there are opportunities to learn how and why these areas are achieving high uptake.

Future childhood and adolescent vaccination programmes can benefit from better understanding how differences in delivery affects uptake. The results of inequalities in the HPV immunisation programme may not appear for a number of years therefore it is essential to understand and acknowledge inequalities in the current programme in order to create and maintain equitable health in every adolescent vaccination programme.

## Supporting Information

Box S1 **Interview questions and topic guide.**
(DOCX)
