# Supplementary material for: Addressing Health Inequalities in the Delivery of the Human Papillomavirus Vaccination Programme: Examining the Role of the School Nurse
Source: PLoS One. 2012 Sep 13;7(9):e43416. doi: 10.1371/journal.pone.0043416 (PMC3441494; doi:10.1371/journal.pone.0043416)
Supplement: Box S1 — Interview questions and topic guide. (DOCX) [file pone.0043416.s001.docx]

Box 1. Interview questions and topic guide

- Outline experience (including job title, years worked on HPV vaccination programme, years worked in area)
- General views on the HPV vaccination programme
- Typical queries from girls and parents (prompt - amount of time this requires, do they have the information to answer these queries)
- Describe how HPV vaccination programme is delivered in their area (prompt - health promotion, consent forms (when distributed, if analysed), administrative support, methods for tracking and monitoring mop-up clinics, opportunistic vaccination, level of support from school, Local Education Authority, Primary Care Trust or Local Authority)
- Official catch-up programme (first three years of programme for older girls) – describe and impact on health inequalities
- Views on why girls were not vaccinated with HPV (prompt - informed decisions by themselves or parents, incomplete consent forms)
- Views on which girls were not vaccinated with HPV (prompt - not attending, vulnerable groups)
- Describe efforts to address health inequalities (prompt - specific reference to travellers, girls with learning difficulties, refugees and asylum seekers, girls not in education or consistently absent and what prompted their efforts)
- Were ethnic / religious groups an issue, specific interventions to address
- How to improve the HPV programme, especially efforts to address health inequalities
